# Supplementary material for: Virion aggregation shapes infection dynamics and evolutionary potential
Source: J Virol. 2025 Oct 15;99(11):e01137-25. doi: 10.1128/jvi.01137-25 (PMC12645961; doi:10.1128/jvi.01137-25)
Supplement: Supplemental figures — Figures S1 to S4. [file jvi.01137-25-s0001.pdf]

Supplementary figures

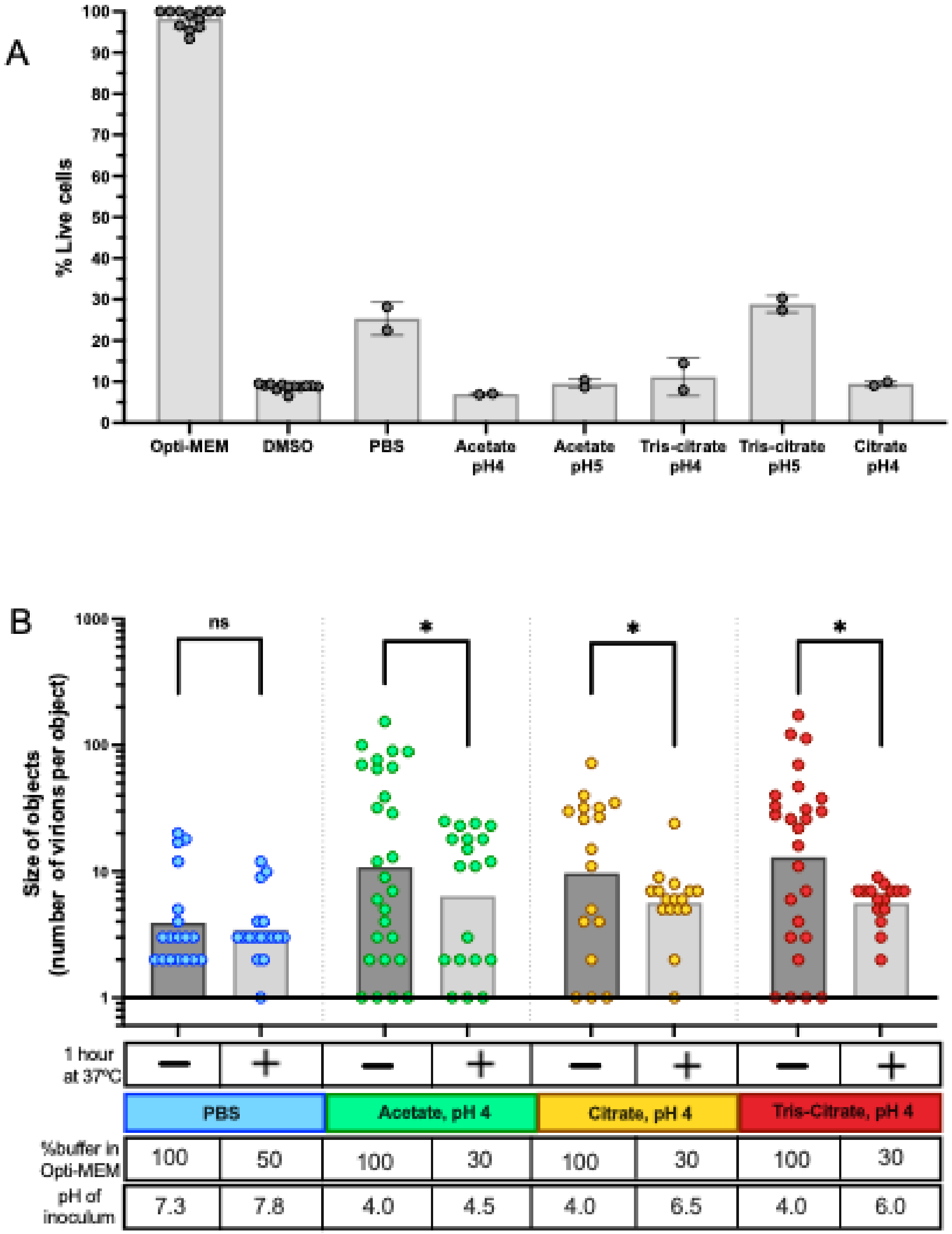

Supplementary Figure 1: *Low pH buffers and PBS are cytotoxic to L929 cells, and reovirus aggregation is reversible and sensitive to buffer pH.* **(A)** Viability of L929 cells was measured in

culture media (Opti-MEM), DMSO, PBS and in various low pH buffers. Live cell percentage was calculated by normalizing the absorbance reading for each condition to that of the Opti-MEM control. DMSO was included as a positive control for cell death. Data represents 6 biological replicates each for Opti-MEM and DMSO and 2 biological replicates for low pH buffers, each with 3 technical replicates. Bars represent the mean ± s.e.m. A Kruskal-Wallis test indicated significant differences across groups (***$p=0.0001$). Pairwise comparisons to Opti-MEM were assessed using two-sided Mann-Whitney U tests, *$p<0.05$. **(B)** Quantification of aggregation levels was measured by dynamic light scattering (DLS), based on the size of viral objects. Treatment conditions are indicated in the table under the plot. Data were pooled from 3 biological replicates. Each data point represents a viral object of size ≥ 1. Bars represent the geometric mean. * $p < 0.03$; ns, non-significant by unpaired Kolmogorov-Smirnov nonparametric test.

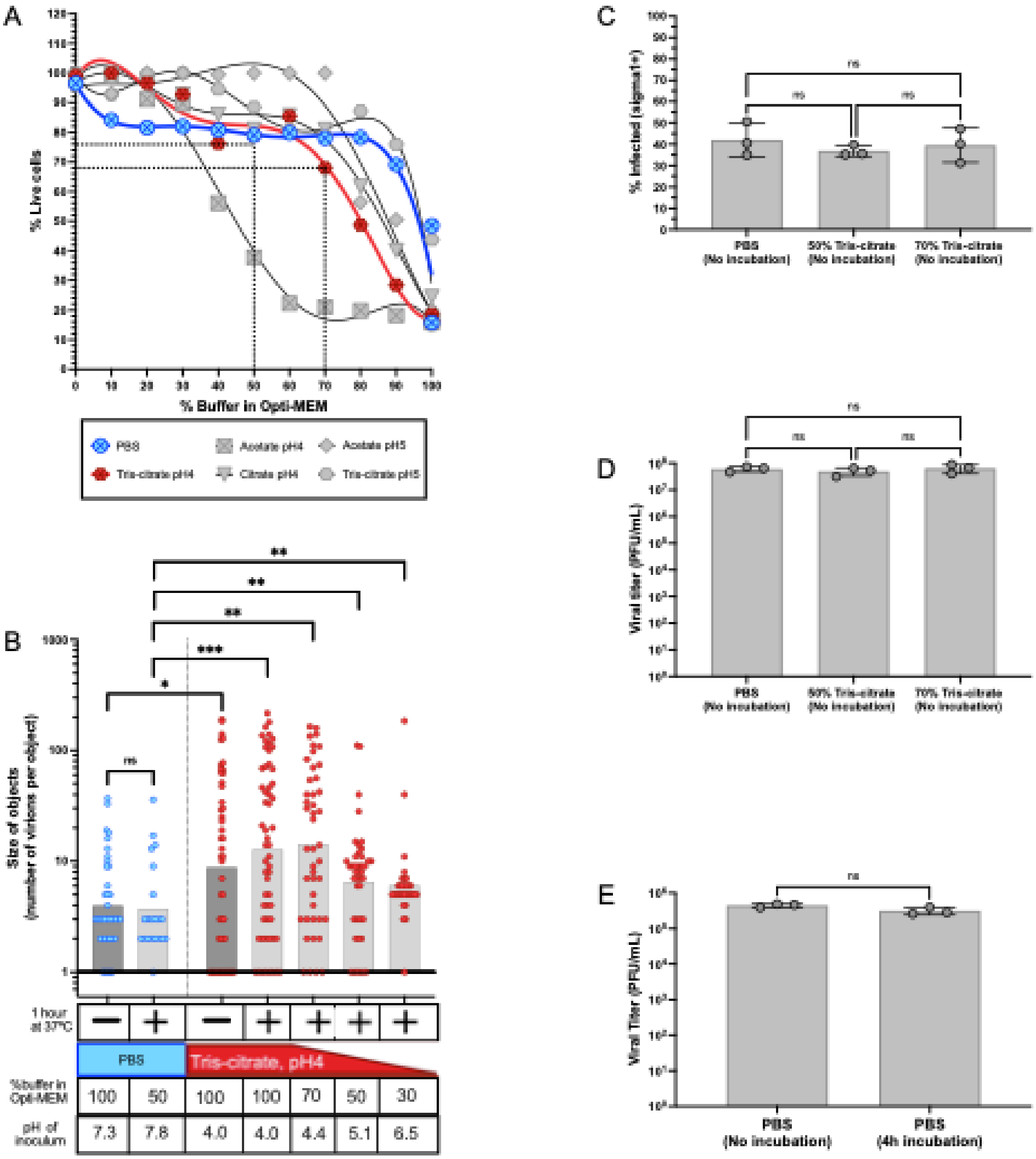

Supplementary Figure 2: *Optimization of aggregation conditions to reduce cellular cytotoxicity.* **(A)** Viability of L929 cells following exposure to PBS and low pH buffers diluted with varying proportions of Opti-MEM culture media, ranging from 10% to 100%. Data represent the mean of 2 biological replicates, each with 2 technical replicates. Nonlinear curve fitting was performed using a fifth-order polynomial regression model. Curves were evaluated using goodness-of-fit (R^2)

values (for all, R^2>0.7, Sys.x <13.64). **(B)** Quantification of aggregation levels measured by DLS, based on the size of viral objects. Data represents 2 biological replicates, each with 3 technical replicates. Each data point represents a viral object of size ≥ 1. Bars represent the geometric mean. **p < 0.0003, **p < 0.003, *p < 0.03 by unpaired Kolmogorov-Smirnov nonparametric test. **(C-D)** Assessing buffer toxicity and viral infectivity of Opti-MEM-buffer mixtures in the absence of aggregation. L929 cells were infected at an MOI of 1 GC/cell and, at 24 h post-infection, the percentage of cells infected was determined by flow cytometry **(C)** and plaque titers were quantified from cell lysates **(D)**. Each data point represents a biological replicate. Bars represent the mean ± s.d. ns, non-significant by ordinary one-way ANOVA followed by Tukey's multiple comparison test. **(E)** Assessing viral infectivity in PBS with and without 4 h incubation. To mimic conditions used for infection experiments, virus in PBS was combined with Opti-MEM following 0 h or 4 h incubation in PBS. Each data point represents an independent biological replicate. Bars represent the mean ± s.d. ns, not significant by unpaired Mann-Whitney test.

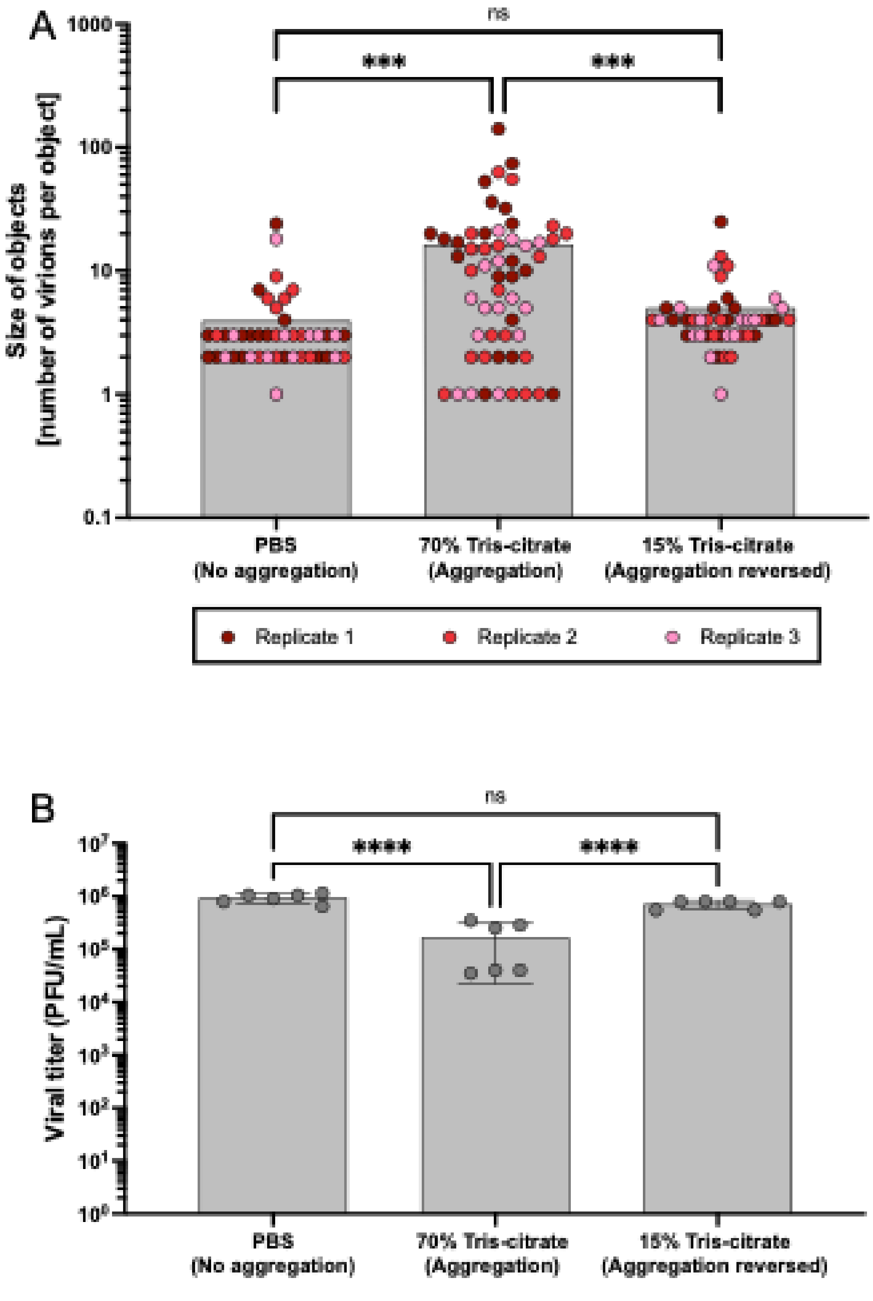

Supplementary Figure 3: *Reovirus is stable in low pH buffers; buffer induced viral aggregation is reversible and pH dependent.* **(A)** Quantification of aggregation levels was performed by measuring the size of viral objects using DLS following treatment with Opti-MEM. Each data point represents an individual viral object of size ≥ 1. Data is pooled from 3 biological replicates. Bars

indicate mean. ****p < 0.0001; ***p < 0.001; ns, not significant by mixed-effects model followed by Tukey's multiple comparison test. **(B)** Infectious titers of virus preparations following treatment with Opti-MEM. Each data point represents an independent biological replicate. Bars represent the mean ± s.d. ****p < 0.0001; ns, not significant by ordinary one-way ANOVA followed by Tukey's multiple comparison test.

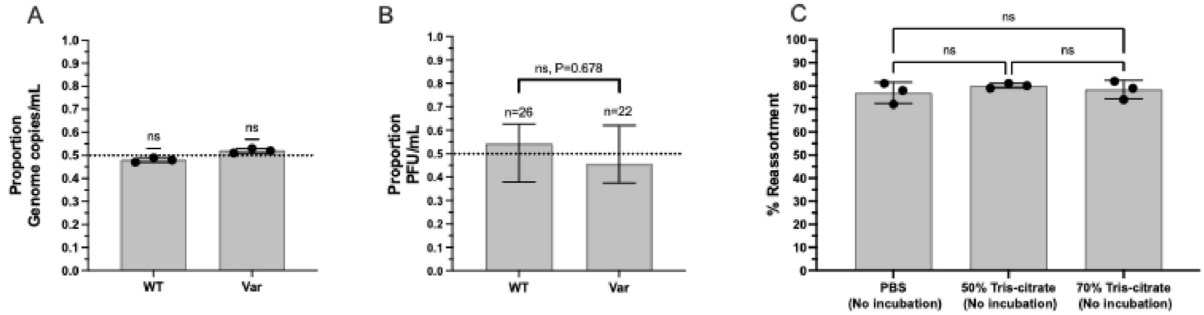

Supplementary Figure 4: *Validation of 1:1 WT-Var mixture and PBS as a negative control for co-infection assays.* **(A)** Validation of 1:1 WT and Var viral mixtures: Genome copy ratios were quantified across 3 biological replicates. Bars represent mean ± 95% confidence interval. Each data point represents a biological replicate. Statistical comparison to the expected proportion of 0.5 was performed by one-sample t-test. ns, non-significant. **(B)** Infectious viral particle ratios were validated by genotyping 48 plaques derived from the mixture of WT and Var viruses. Bars represent mean ± 95% confidence interval. A two-sided exact binomial test was used to determine whether the proportions of WT and Var differ significantly (p=0.678) and 95% confidence intervals were calculated using Clopper-Pearson method. **(C)** Reassortment frequency in L929 cells co-infected with WT and Var reoviruses prepared in the indicated buffers without incubation to induce aggregation. MOI was 1 GC/cell. Data shown are mean ± s.d. and represent 3 biological replicates. Each data point represents the percentage of reassortant plaques out of 32 plaques. ns, not significant by ordinary one-way ANOVA followed by Tukey's multiple comparison test.
